# Post Traumatic Lemmel’s Syndrome: A Rarest of Rare Cause of Obstructive Jaundice

**DOI:** 10.7759/cureus.9862

**Published:** 2020-08-19

**Authors:** Kunal Parasar, Aaron G John, Shantam Mohan, Utpal Anand

**Affiliations:** 1 Surgical Gastroenterology, All India Institute of Medical Sciences, Patna, IND; 2 Gastroenterology, All India Institute of Medical Sciences, Patna, IND

**Keywords:** lemmel’s syndrome, post traumatic obstructive jaundice, post traumatic lemmel’s syndrome

## Abstract

Obstructive jaundice caused by periampullary duodenal diverticulum in absence of choledocholithiasis or tumor is known as Lemmel syndrome. This is a rare cause of obstructive jaundice. We report here a patient of blunt trauma abdomen who underwent emergency laparotomy whose sequelae was a controlled external biliary fistula which healed and led to obstructive jaundice. What appeared to be a clear cut diagnosis of benign biliary stricture or bilioma gave a surgical surprise on opening the pandoras box. The uniqueness of this case lies in its etiopathogenesis as well as the dearth of available literature related to post traumatic Lemmel syndrome. This case provides us with a insight into an easy to be overlooked cause of obstructive jaundice in the absence of duodenal diverticula.

## Introduction

Obstructive jaundice is a common presentation in surgical wards with choledocholithiasis and hepatobiliary malignancies being the most common causes. Nevertheless, there are other rare differential diagnoses that sometimes need to be considered [1]. Herein, we present a case of a particularly rare cause of obstructive jaundice in its rarest presentation.

## Case presentation

A 21-year-old, healthy male patient was admitted in a peripheral center with blunt trauma to the abdomen. He presented with tachycardia and abdominal distension. Ultrasound abdomen showed a large collection in the peritoneal cavity. On performing exploratory laparotomy, more than 3 liters of blood mixed bilious fluid was present. There was a 4x4 cm hematoma seen in segment 5 and 8 of the liver. Since the source of bile leak could not be localized intraoperatively, a wide bore drain was placed. In the post-operative period, he continued to have significant drain output (≈ 600 ml) per day which was bilious in nature and was referred to our center. With the above history and clinical presentation and taking into account the patient’s condition, he was managed as a case of controlled external biliary fistula. During the hospital stay, the drain output gradually decreased and the drain was removed after 29 days of primary surgery.

**Figure 1:**
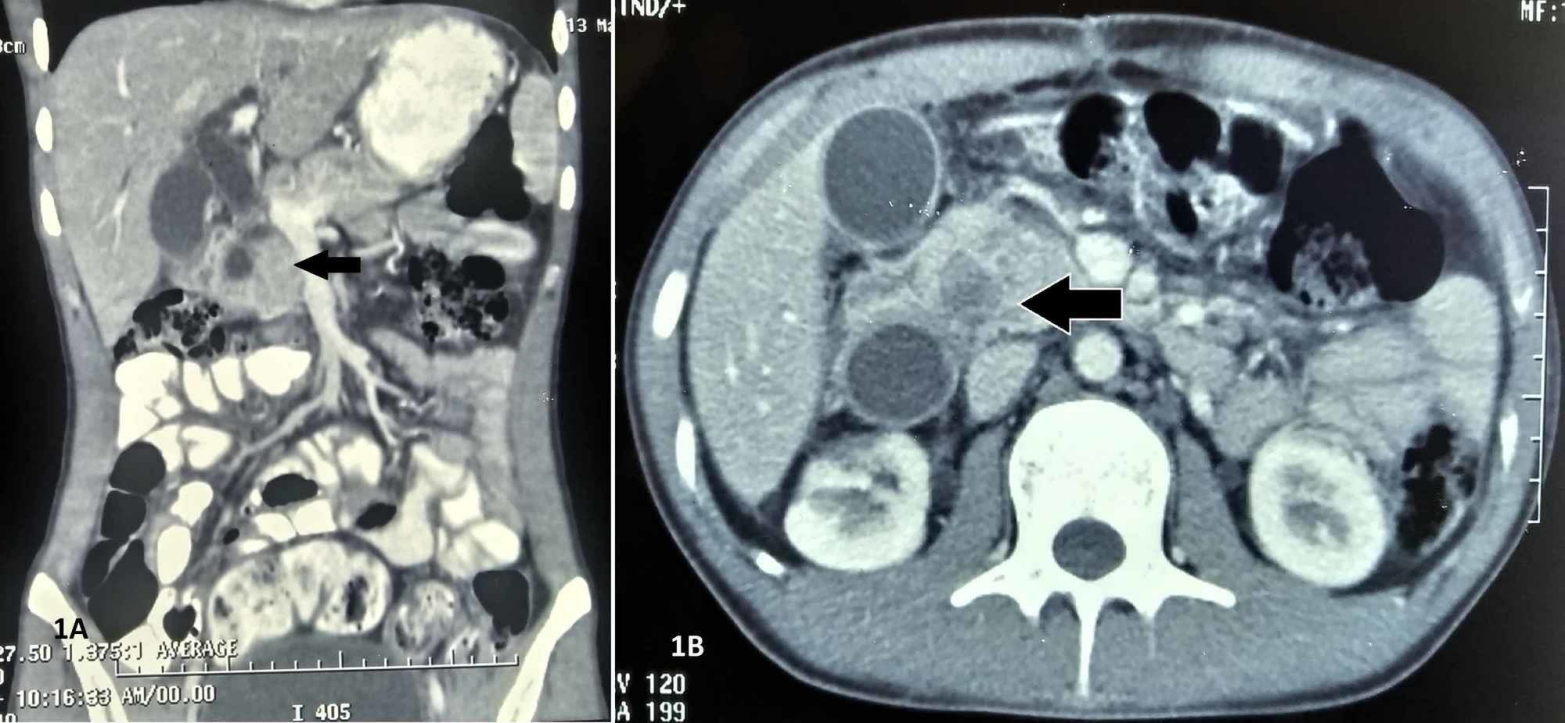
Focal out pouching of the duodenum causing compression of common bile duct. A: coronal cut. B: axial cut.

Two weeks after drain removal, he developed painless and progressive cholestatic jaundice. Total Bilirubin was 12.6 mg/dl and serum alkaline phosphatase was 740 IU/L. Contrast enhancing CT scan showed a 3x3 cm heterogenous cystic lesion compressing the supraduodenal part of common bile duct (CBD) with non-visualized distal duct associated with the presence of debris inside the cyst (Figure 1). Proximal CBD was dilated with bilobar intrahepatic biliary radicle dilatation.

**Figure 2:**
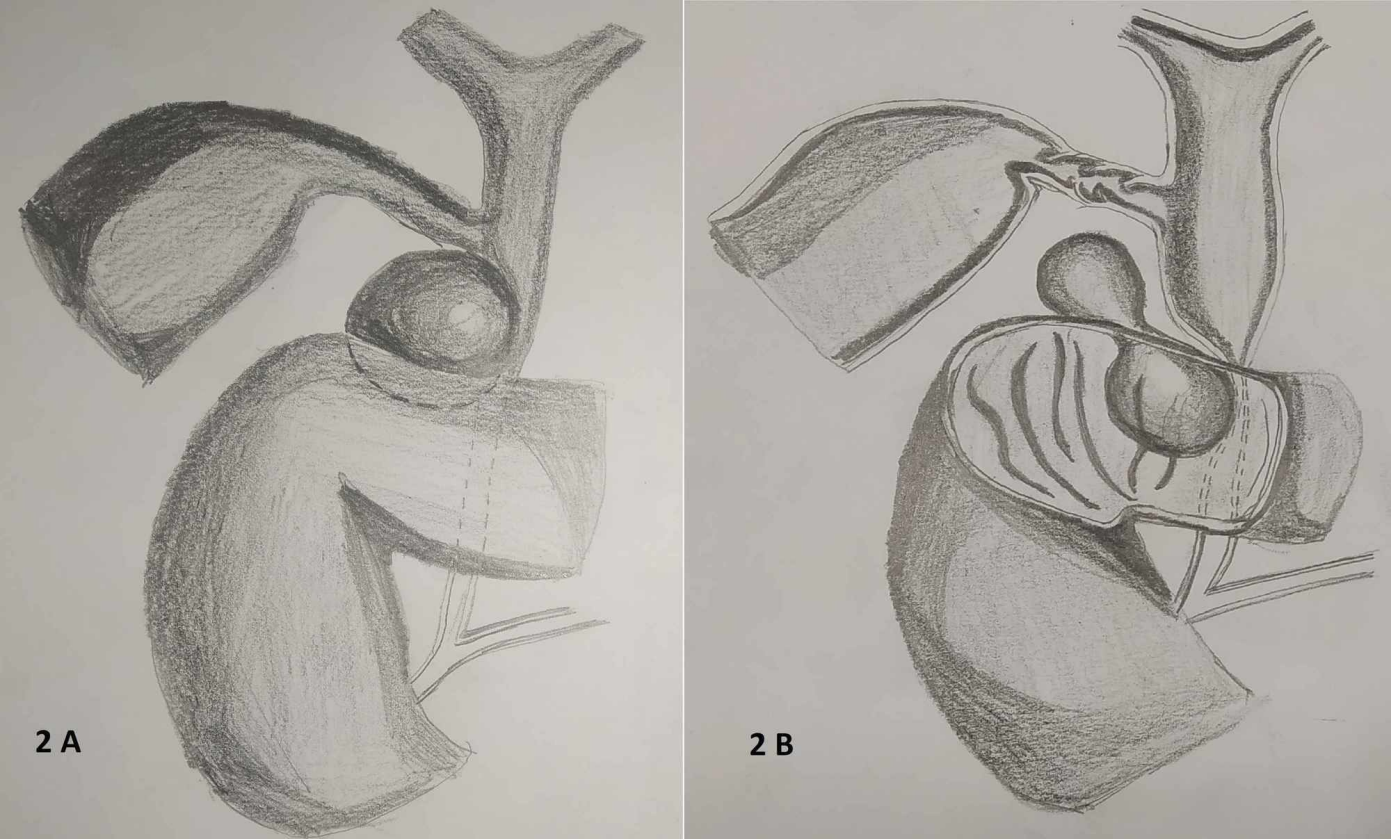
The expected and the unexpected. (A) shows what we were anticipating, an extrinsic diverticulum compressing the common bile duct. (B) shows the intraoperative finding. (Freehand illustration of the intraoperative finding.)

Initially, drainage under radiological guidance was planned but taking into consideration the presence of debris, we intervened surgically. Intraoperatively, there were dense perihepatic adhesions. Post adhesiolysis, a cystic lesion with a well-formed wall was located on posterosuperior wall of first part of duodenum and was not adherent to lower CBD (Figure 2B).

It was not possible to kocherize the duodenum without dissecting the cyst. The cyst was meticulously dissected and duodenum was kocherized retrograde. The cyst was originating from first part of duodenum. The cyst was incised draining dirty greenish bile with debris. Cyst wall was excised and primary closure of duodenum was done. Roux-en-Y choledochojejunostomy was done. His post-operative course was uneventful and was discharged on fifth postoperative day. At the time of writing he remains asymptomatic.

We did an exhaustive search of available literature and came across Lemmel’s syndrome. It has been reported very rarely in literature and post traumatic Lemmel’s syndrome has never been reported.

## Discussion

Duodenal diverticulas (DD) have been detected in 12%-27 % of endoscopies and 15%-23 % of autopsies. The exact incidence is unknown since the majority remain asymptomatic [2]. They were first described in 1710 by the French pathologist Chomel. Seventy-five per cent are juxtapapillary in location, occurring within 3 cm of the ampulla. It develops due to mucosal herniation through a defect in the muscular layer, therefore its incidence increases with age [1].

Lemmel’s syndrome is defined as obstructive jaundice due to juxtapapillary diverticulum in the absence of cholelithiasis or other detectable obstacle, but to date only few cases have been published. Patients may present with chronic abdominal pain, gastrointestinal bleeding, diverticulitis, perforation, intestinal obstruction and intermittent jaundice [3].

There are many theories regarding the pathophysiology of this condition. Mechanical irritation of periampullary diverticula may lead to chronic inflammation of the ampulla, which causes fibrosis of the papilla. Periampullary diverticula may cause dysfunction of the sphincter of Oddi. Distal common bile duct or ampulla can be compressed mechanically by periampullary diverticula. The obstruction increases the incidence of choledocholithiasis due colonization and overgrowth of β-glucuronidase producing bacteria, which deconjugates bilirubin glucuronides and precipitates calcium bilirubinate gallstones [4,5].

The diagnosis is confirmed by imaging, which reveals a lateral compression of the distal CBD by the diverticulum. The uniqueness was the sequence of events that masqueraded as benign biliary stricture or bilioma leading to biliary obstruction. Endoscopic sphincterotomy is considered as the first treatment for symptomatic patients. It might be successful in most patients but the recurrence rates are reportedly high. Surgical interventions are deferred till endoscopic options have been exhausted. An end-to-side Roux-en-Y choledochojejunostomy is often preferred [6]. Transduodenal sphincteroplasty is another option.

The importance of this case lies in its bizarre clinical presentation. This case had all the preoperative evidence of traumatic biliary stricture. What we witnessed, was an intraoperative surprise. The chronology of events in our case most likely was; during blunt trauma abdomen patient had a small injury to the posterior side of first part of duodenum near the retroduodenal CBD which led to the formation of a massive bilioma. Due to it being a small rent lying at junctional site, it was missed during exploratory laparotomy. Gradually the injury would have healed by formation of pseudocyst, which contained debris. The cyst had both an extraduodenal and an intraduodenal component. Imaging showed extraduodenal component compressing supraduodenal CBD (Figure 1). Instead what came, as the intraoperative surprise was that the obstruction was due to the intraduodenal cystic component, which was not visualized in the imaging and led to obstruction of retroduodenal CBD causing obstructive jaundice (Figure 2B).

We were fortunate that the supra duodenal cyst was not in close contact with CBD, leading us to further dissect the cyst to look for the site of obstruction. Had it been in close contact with duct, we may have missed the actual site of obstruction considering it to be consistent with our preoperative diagnosis. Even though such cases are rare we must be aware of them, so that they can be managed confidently improving patient outcome.

## Conclusions

The importance of this case lies in its bizarre clinical presentation. This case had all the preoperative evidence of traumatic biliary stricture. What we witnessed, was an intraoperative surprise. We were fortunate that the supra duodenal cyst was not in close contact with CBD, leading us to further dissect the cyst to look for the site of obstruction. Had it been in close contact with duct, we may have missed the actual site of obstruction considering it to be consistent with our preoperative diagnosis. Even though such cases are rare we must be aware of them, so that they can be managed confidently improving patient outcome.

## References

[1] Rouet J, Gaujoux S, Ronot M (2012). Lemmel’s syndrome as a rare cause of obstructive jaundice. Clin Res Hepatol Gastroenterol.

[2] Teven CM, Grossman E, Roggin KK, Matthews JB (2012). Surgical management of pancreaticobiliary disease associated with juxtapapillary duodenal diverticula: case series and review of the literature. J Gastrointest Surg.

[3] Medina Andrade L (2016). Cholangitis secondary to Lemmel syndrome: case report. Arch Clin Gastroenterol.

[4] Kang HS, Hyun JJ, Kim SY (2014). Lemmel’s syndrome, an unusual cause of abdominal pain and jaundice by impacted intradiverticular enterolith: case report. J Korean Med Sci.

[5] Desai K, Wermers JD, Beteselassie N (2017). Lemmel syndrome secondary to duodenal diverticulitis: a case report. Cureus.

[6] Manny J, Muga M, Eyal Z (1981). The continuing clinical enigma of duodenal diverticulum. Am J Surg.

